# Core and skin temperature influences on the surface electromyographic responses to an isometric force and position task

**DOI:** 10.1371/journal.pone.0195219

**Published:** 2018-03-29

**Authors:** Nico A. Coletta, Matthew M. Mallette, David A. Gabriel, Christopher J. Tyler, Stephen S. Cheung

**Affiliations:** 1 Department of Kinesiology, Brock University, St. Catharines, Ontario, Canada; 2 Department of Life Sciences, University of Roehampton, London, United Kingdom; Universite de Nantes, FRANCE

## Abstract

The large body of work demonstrating hyperthermic impairment of neuromuscular function has utilized maximal isometric contractions, but extrapolating these findings to whole-body exercise and submaximal, dynamic contractions may be problematic. We isolated and compared core and skin temperature influences on an isometric force task versus a position task requiring dynamic maintenance of joint angle. Surface electromyography (sEMG) was measured on the flexor carpi radialis at 60% of baseline maximal voluntary contraction while either pushing against a rigid restraint (force task) or while maintaining a constant wrist angle and supporting an equivalent inertial load (position task). Twenty participants performed each task at 0.5°C rectal temperature (T_re_) intervals while being passively heated from 37.1±0.3°C to ≥1.5°C T_re_ and then cooled to 37.8±0.3°C, permitting separate analyses of core versus skin temperature influences. During a 3-s contraction, trend analysis revealed a quadratic trend that peaked during hyperthermia for root-mean-square (RMS) amplitude during the force task. In contrast, RMS amplitude during the position task remained stable with passive heating, then rapidly increased with the initial decrease in skin temperature at the onset of passive cooling (*p* = 0.010). Combined hot core and hot skin elicited shifts toward higher frequencies in the sEMG signal during the force task (*p* = 0.003), whereas inconsistent changes in the frequency spectra occurred for the position task. Based on the patterns of RMS amplitude in response to thermal stress, we conclude that core temperature was the primary thermal afferent influencing neuromuscular response during a submaximal force task, with minimal input from skin temperature. However, skin temperature was the primary thermal afferent during a position task with minimal core temperature influence. Therefore, temperature has a task-dependent impact on neuromuscular responses.

## Introduction

High ambient temperatures impair whole-body exercise tolerance [1] and reduce self-paced exercise intensity [2]. Multiple mechanisms have been advanced for this premature fatigue and reduced exercise capacity, including that sustained heat storage and hyperthermia directly causes a decrement in neuromuscular function [3]. Prior work, predominantly performed using isometric, maximal single-joint muscle contractions has endorsed a direct hyperthermic impairment on central nervous system activation [4–7]. In these studies, voluntary activation progressively decreased with higher core temperature and then progressively increased back towards baseline levels only upon subsequent core cooling, with these changes in voluntary activation being independent of changes in skin [4] and muscle temperature [6].

However, extrapolating core temperature impairment during isometric and maximal contractions to whole-body exercise may be problematic [8]. While maximal isometric contractions provide an understanding of the force producing capacity of the neuromuscular system [9], neural activation patterns of maximal isometric contractions are poorly related to dynamic movements such as sprinting and vertical jump test performance [10]. Rather, isotonic contractions–which inherently demonstrate lower electromyographic activity [11], lower motor unit discharge rate [12], recruitment of additional motor units during prolonged contractions [13,14], and greater rate of increase in central neural activity [11,12] compared to isometric contractions–may have greater neural and mechanical similarities to dynamic movements typical of whole-body exercise [15,16]. Dynamically maintaining position of a joint against a specified load with a consistent muscle tension–often termed an iso-inertial contraction–is neurologically similar to an isotonic contraction, and may be a useful analog for studying dynamic muscle movements.

Differences in how core and skin thermal stimuli affect different neuromuscular tasks have been previously reported [4,6,8,17–21]. Contrary to observations during isometric contractions, maximal dynamic isokinetic contractions appear to be unaffected by rising core temperature; rather, lowering skin temperature immediately impaired force production prior to any reduction in core temperature, with this impairment sustained throughout the period of core temperature decreasing back to baseline [22]. These data suggest that neuromuscular function during hyperthermia is dependent on both the source of thermal stimuli and the type of task being performed. Surface electromyograpy (sEMG) is a non-invasive method to objectively quantify neuromuscular activation of muscle [23], and could be a valuable technique to investigate the physiological mechanisms responsible for these differences. Additionally, sEMG has been used extensively to investigate various neuromuscular disorders [24], muscular fatigue [9,25–27], and the effects of temperature on muscle properties [28,29]. Yet, none of the previous works have directly compared the effects of core and skin temperature on different neuromuscular tasks, nor investigated the contribution of thermal afferents while using sEMG.

Therefore, the purpose of this experiment was to compare the effect of high core versus skin temperature on the neuromuscular response to static and dynamic muscle contractions. Submaximal (60% of maximal voluntary contraction [MVC]) isometric force and position tasks were used as a surrogate for static and dynamic muscle contractions, respectively, because they are biomechanically comparable with respect to changes in muscle length and moment arm versus a freely movable limb while maintaining a constant muscle tension. The force (isometric) task consisted of wrist flexion against an immovable brace, while the position (isotonic) task–where the force requirements are the same, and the limb is free to move—required dynamically maintaining the wrist in a stable position against 60% of baseline maximal force. Testing was performed at core temperature intervals of 0.5°C during heating to ≥ 1.5°C above baseline core temperature, and then during cooling back to baseline in order to test the relative contributions of core versus skin temperatures on neural drive. It was hypothesized that core temperature would be the primary influence on sEMG magnitude and frequency changes during the isometric force task, while skin temperature would be the primary influence on these variables during the isometric position task.

## Methods

### Participants

Twenty healthy individuals (13 males and 7 females) were recruited. The mean (± SD) age, height, mass, and body fat percentage was 23.8 ± 2.1 years, 175.3 ± 8.5 cm, 70.4 ± 9.2 kg, and 14.8 ± 6.0%, respectively. The study was approved by the Bioscience Research Ethics Board of Brock University (REB 15–076) and conformed to the standards set by the Declaration of Helsinki. All participants were screened for cardiovascular and neuromuscular health using the Physical Activity Readiness Questionnaire developed by the Canadian Society for Exercise Physiology, then were informed of the experimental protocol and associated risks prior to providing verbal and written informed consent.

### Familiarization session

Participants completed a familiarization session to acquaint themselves with the apparatus and to practice the experimental protocol. During this session, height (cm) and mass (kg) were determined using standard laboratory equipment. Body fat percentage (%) was determined through a 7-site (triceps, sub-scapula, abdomen, supra-iliac crest, mid-axilla, thigh, and pectoralis major) skinfold thickness [30,31] with manual calipers (Harpenden, Bay International, West Sussex, UK).

### Experimental protocol

The study was a repeated measures design occurring over a single session, with neuromuscular testing taking place during passive heating from baseline to voluntary tolerance, which was an increase of at least 1.5°C rectal temperature (T_re_), followed by passive cooling back towards baseline T_re_. Female participants were in the early follicular phase of their menstrual cycle as determined through self-report to control for differences in initial T_re_. Prior to the experimental session, participants were asked to abstain from strenuous exercise and the consumption of caffeine for 12 h and alcohol for 24 h. Upon arrival to the laboratory (~22°C, ~43% relative humidity) between 0800–1300 h for the experimental session, participants voided their bladder and euhydration was confirmed using a refractometer (PAL-10S, Atago, USA) and a threshold urine specific gravity ≤ 1.020 [32].

Participants self-inserted a thermistor (Mon-A-Therm Core, Mallinkrodt Medical, St. Louis, USA) 12 cm beyond the anal sphincter to measure T_re_. Thermocouples (PVC-T-24-190, Omega Environmental Inc. Laval, QC, Canada) were taped at four sites to calculate mean skin temperature (${\bar{T}}_{\mathrm{sk}}$) using a weighted average of 0.3(chest) + 0.3(arm) + 0.2(thigh) + 0.2(calf) [33].

Participants were dressed in a three-piece liquid-conditioning garment (BCS 4 Cooling System, Allen Vanguard, Ottawa, CAN) consisting of 1/8” Tygon tubing sewn into a stretchable suit. The liquid-conditioning garment covered the arms, upper and lower legs, and torso; the face, head, neck, hands, and feet were left uncovered. Males wore shorts, while females wore shorts and a sports bra. To minimize evaporative heat loss, an impermeable polyvinyl rain suit was worn overtop the liquid-conditioning garment. Passive hyperthermia–target of a minimum 1.5°C ΔT_re_ above baseline–was performed by adding 49.5°C water maintained by a temperature controller (Model 5202, Polyscience, Niles, IL, USA) and pumped (MED-ENG, Pembroke, CAN) at a flow rate of ~1.5 L·min-1. This was then followed by passive cooling, with 10°C water flowing through the liquid-conditioning garment until T_re_ decreased to 1.0°C above baseline or lower.

**Fig 1:**
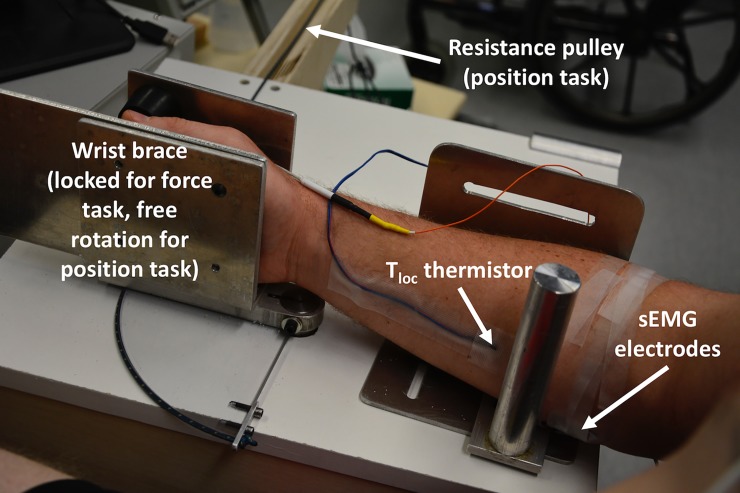
Arm brace setup. Brace isolating the flexor carpi radialis muscle during wrist flexion for both the isometric force and position tasks, illustrating the skin temperature thermistor for local forearm temperature (T_loc_) and sEMG electrodes. Resistance for the position task provided by weights suspended on a pulley at the end of the wooden arm.

To isolate wrist flexion and the flexor carpi radialis, participants were positioned in a semi-recumbent position on an examination table with their right forearm supported at approximately 135° elbow extension in a custom-made apparatus (Fig 1). For the force task, the fingers were extended and the carpals to distal phalanges of the right hand were placed between two fixed aluminum plates, with the right forearm positioned such that the styloid process was aligned with the axis of rotation. These plates were secured to a calibrated load cell and potentiometer to measure torque and angular displacement of the wrist joint, respectively. For calibration, a known weight was attached to the load cell and the voltage output (torque acquired through A-D converter @ 2500Hz) viewed; voltages outputs for weight below expected force to above expected force were used to build a linear regression equation.

Participants were tasked with performing wrist flexion against the fixed plates, and viewed a monitor that displayed the target torque. For the position task, the same elbow, forearm, and wrist support occurred, but the aluminum plates bracing the hand were not fixed, such that wrist flexion and extension were enabled. Participants were tasked with dynamically maintaining the wrist in a neutral position against a load that forced wrist extension, while the monitor provided information on angular displacement.

### Neuromuscular measurements

**Fig 2:**
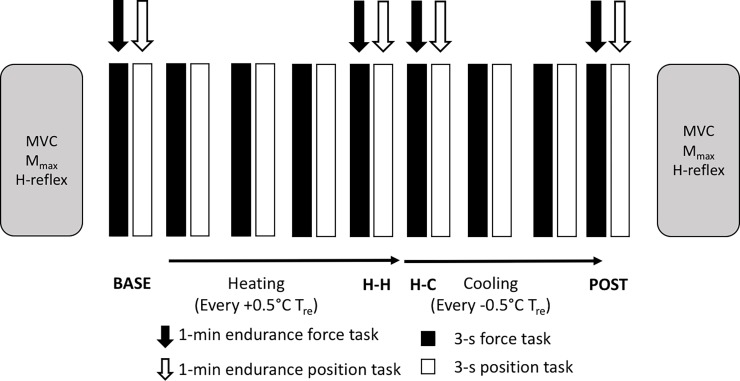
Schematic of the experimental timeline, outlining the neuromuscular test battery along with the heating and cooling protocol. BASE: testing at baseline prior to passive heating; H-H: Hot core, hot skin, taken at the highest point of T_re_ rise tolerated (>1.5°C T_re_ for all participants); H-C: Hot core, cool skin, taken <5 min after initiation of cooling; POST: Neutral core, cool skin, taken at the end of cooling after T_re_ returned to <+1.5°C from BASE.

The heating and cooling protocol, along with timing of neuromuscular testing, is outlined in Fig 2. Neuromuscular testing before (PRE) and following (POST) the passive heating and cooling protocol consisted of eliciting five maximal M-wave (M_max_) and five H-reflex responses through electrical stimulation of the median nerve, each separated by 15 s. Subsequently, participants performed five maximal voluntary isometric contractions (MVC); each contraction was 5 s in duration separated by 2 min. These were performed to test for whether the contractions performed over the course of the experiment, or the heating and cooling protocol itself, induced significant fatigue. The highest MVC pre-heating was used to calculate the 60% MVC force output required during the subsequent submaximal force and position tasks.

During the passive heating and cooling protocol, participants performed a set of 60% MVC force and position tasks at each 0.5°C T_re_ interval; each task was 3 s in duration and consisted of two submaximal isometric force tasks followed by two position tasks (the order of contractions was counterbalanced across individuals). Participants also performed a single submaximal (60% of MVC) isometric force and position tasks for 1 min at each of four distinct temperature states: initial T_re_ and ${\bar{T}}_{\mathrm{sk}}$ (BASE); hot T_re_, hot ${\bar{T}}_{\mathrm{sk}}$ (H-H) after passive heating of T_re_ ≥ 1.5°C above baseline; hot T_re_, cool ${\bar{T}}_{\mathrm{sk}}$ (H-C) shortly after cold (10°C) water began circulating through the liquid-conditioning garment; and the end of the protocol where T_re_ returned to near baseline and ${\bar{T}}_{\mathrm{sk}}$ was cool (POST). A 1 min, 60% MVC was used in this study because this was the highest submaximal contraction that could be sustained and repeated without evoking fatigue. This was demonstrated through no changes in root-mean-square (RMS) amplitude in the sEMG signal before and after the heating protocol.

### Methodological controls

Pilot testing was completed to determine the effect of time, boredom, and potential fatigue on neuromuscular function. Three participants completed a control trial during which they performed the experimental protocol without any thermal manipulations, with one participant fully instrumented with sEMG. The control trial lasted 2 h, with participants performing submaximal contractions every 20 min; no differences in torque output or EMG parameters were observed.

Previous work [34] has shown that stabilization of the force and sEMG trace is achieved within 10 contractions. Therefore, at the familiarization session for all participants, maximal contractions were practiced until a plateau was achieved, and the 60% force and positions tasks were practiced 10 times. In order to reduce the likelihood of fatigue occurring during the familiarization session, we used shorter 10–20 s practice trials.

For the experimental session for all participants, MVCs and evoked potentials before (PRE) and following (POST) the passive heating and cooling protocol served as further control, to determine that any neuromuscular changes that occurred during the experimental protocol were a result of thermal manipulations rather than the protocol itself.

### Electromyography

Flexor carpi radialis (FCR) muscle sEMG was measured using pediatric-sized surface electrodes (3 mm electrode diameter, F-E9M 11 mm, GRASS Technologies, Astro-Med, Inc., Warwick, USA) with an inter-electrode distance of 10 mm. To ensure skin-electrode impedance below 10 kΩ, the skin surface was shaved and abraded with electrolyte gel (NuPrep®, Weaver and Company, Aurora, USA) and cleansed with isopropyl alcohol pads (Dukal Corporation, Ronkonkoma, USA). The motor point of the FCR was located by applying a low-level electrical stimulus to the muscle belly. The point that elicited the largest sEMG response at the lowest stimulation intensity was identified as the motor point. Electrodes were placed in a bipolar electrode configuration, as it is reliable for both evoked and voluntary measures [23], one on the electrically identified motor point, and the other placed immediately distal (10 mm). The electrodes were secured to the skin surface with two-sided tape and electrolyte gel (Signa Gel®, Parker Laboratories, Fairfield, USA). The electrodes were then taped (Transpore™, 3M, St. Paul, USA) to ensure contact with the skin throughout the experimental protocol. A 100 mm circular self-adhesive ground electrode (Dermatrode, Delsys, Boston, USA) was placed on the dorsal side (back) of the hand for electrical safety and to minimize noise.

### Evoked potentials

Placed medial to the elbow crease, the anode and cathode were connected in series with an isolation unit (Grass Telefactor SIU8, Astro-Med Inc., Warwick, USA) and constant current stimulator (Grass Telefactor S88, Astro-Med Inc., Warwick, USA) that delivered a square-wave pulse 0.5 ms in duration. Median nerve stimulation was progressively increased until reaching a plateau in the peak-to-peak M-wave amplitude (M_max_). The H-reflex was elicited using low-level electrical stimulation until reaching peak-to-peak amplitude of 10% of M_max_.

### Data processing

Surface EMG signals were amplified (Grass P511, Astro-Med, Inc., Warwick, USA) and band-pass filtered between 3 and 1000 Hz. Torque, sEMG, and displacement signals were acquired at a sampling rate of 2500 Hz using an analog to digital converter (model BNC-2110, National Instruments, Austin, USA), and simultaneously recorded on a personal computer using custom-designed software (LabView 2011, National Instruments, Austin, USA). Torque was low-passed filtered (24 Hz, 3 dB) using a 2^nd^ order Butterworth digital filter offline in MATLAB® (The Mathworks Inc., Natick, USA). Temperature (T_re_, ${\bar{T}}_{\mathrm{sk}}$, T_loc_) data were collected at 1 Hz and electrocardiogram at 1000 Hz (PowerLab, ADInstruments, Colorado Springs, USA), and stored on a personal computer to be analyzed and processed offline using LabChart (Version 8, ADInstruments, Colorado Springs, USA).

For the brief, 3-s contractions, data were obtained from the average of two contractions for both tasks. Eight participants reached their thermal tolerance at +1.5°C T_re_ and their data were grouped in the ‘H-H’ condition. Due to the unequal sample size beyond +1.5°C T_re_ and to maintain consistency between contraction types (3-s and 1-min), the +1.5°C condition was not used for analysis. As the 3-s contractions were not performed at BASE, H-H, H-C, and POST timepoints, the first 3-s of the 1-min contractions were used for these thermal states. Data for the 1-min contraction were obtained for the mean of 4, 1-s windows at 14, 29, 44, and 59-s.

### Statistical analysis

Normal distribution was assessed by skewness and kurtosis measures, and by visual inspection of histograms. Normality was defined as skewness and kurtosis less than ± 3 and ± 9, respectively. Results are presented in mean ± SD with sample size (*n*). Two participants were excluded from the 1 min tasks as they were not able to complete the full min of contraction, therefore a sample size of 18 was used. Paired samples *t*-tests were performed to compare torque and sensorimotor responses before (PRE) and following (POST) the passive heating and cooling protocol to determine whether overall fatigue from the thermal manipulation or repeated contractions affected responses. The primary comparisons of interest were the patterns of responses of the force and position tasks to the heating and cooling protocol. All sEMG data collected were analyzed with a 2-way (T_re_ x contraction type) repeated measures analysis of variance (ANOVA). Bonferonni *post-hoc* corrections were performed for multiple comparisons where significant main effects were found. Complex interactions were explored using orthogonal polynomials to evaluate trends within the data, and were performed using SPSS 23 (SPSS Inc., Chicago, USA). Paired samples *t*-tests and repeated measures ANOVAs were performed with GraphPad Prism (version 7.0, GraphPad Software Inc., La Jolla, USA).

## Results

### Thermal manipulation

**Fig 3:**
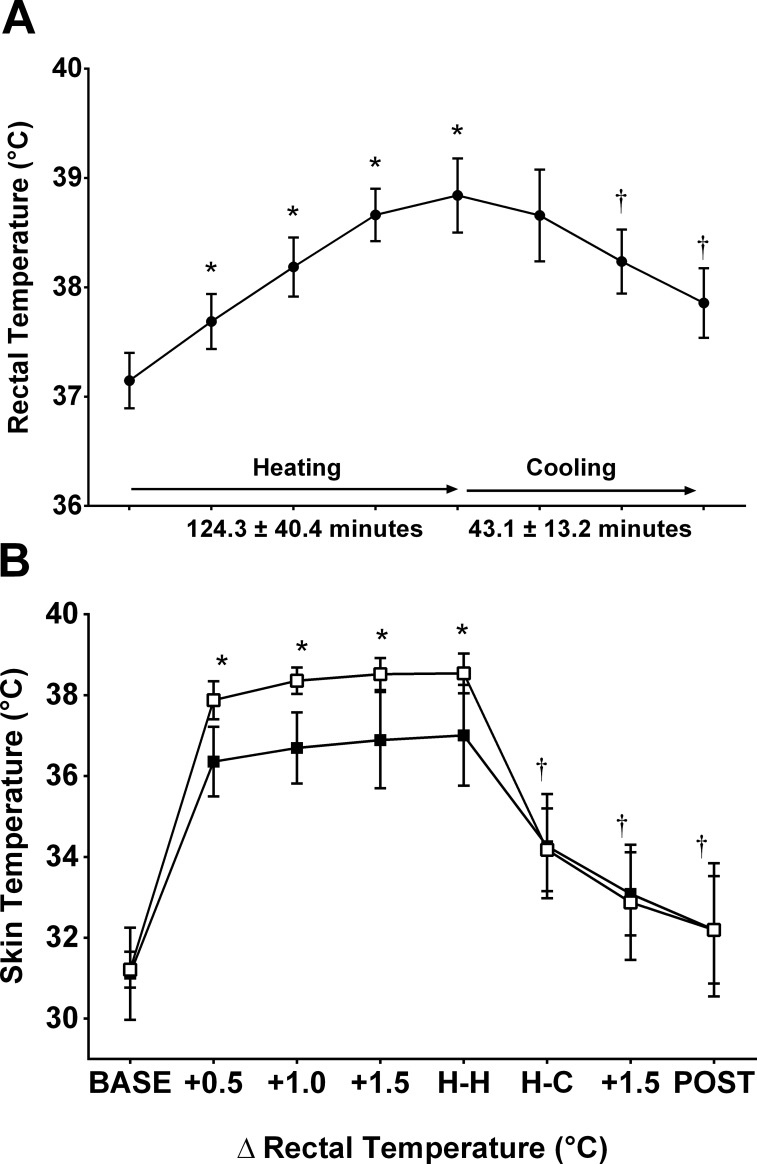
**Rectal temperature (A) and skin temperature responses (B) of mean skin (open squares) and local forearm temperature (closed squares) responses to the passive heating and cooling protocol.** *Significantly different versus initial T_re_ and $\bar{T}\mathrm{sk}$ (BASE; *p* < 0.001). †Significantly different versus hot T_re_, hot ${\bar{T}}_{\mathrm{sk}}$ (H-H; *p* < 0.001).

The protocol was successful in eliciting the desired manipulations of T_re_ and ${\bar{T}}_{\mathrm{sk}}$ (Fig 3, S1 File). During passive heating, T_re_ increased > 1.5°C for all participants (*p* < 0.001, *n* = 20), from 37.1 ± 0.3°C at baseline to 38.8 ± 0.3°C; 8 of the 20 participants terminated heating at this point, while the remaining 12 tolerated 1.9–2.1°C increase in T_re_. The point at which participants reached their highest T_re_ during the heating phase was defined as ‘H-H’. ${\bar{T}}_{\mathrm{sk}}$ was increased (*p* < 0.001, *n* = 20) from 31.2 ± 0.4°C to 38.5 ± 0.5°C during passive heating (H-H), and was rapidly cooled to 34.1 ± 1.1°C while T_re_ remained at 38.7 ± 0.4°C (H-C).

### Methodological controls

Maximal voluntary torque and evoked potential responses at baseline (PRE) and following (POST) the passive heating and cooling protocol are outlined in Table 1. All values were similar PRE and POST. V-wave peak-to-peak amplitude was also similar for both contraction types with four participants (*p* > 0.050, *n* = 4).

**Table 1:** Maximal voluntary contraction, M wave and H-reflex amplitudes before (PRE) and following (POST) the heating and cooling protocol.

	PRE	POST	*P* value
**MVC (N·m^-1^)**	28.5 ± 11.2	27.8 ± 10.6	0.42
**Peak-to-peak M_max_ (mV)**	4.6 ± 1.7	4.5 ± 1.8	0.27
**H-reflex amplitude (mV)**	0.39 ± 0.17	0.40 ± 0.16	0.50

### Brief 3-s force and position task

**Fig 4:**
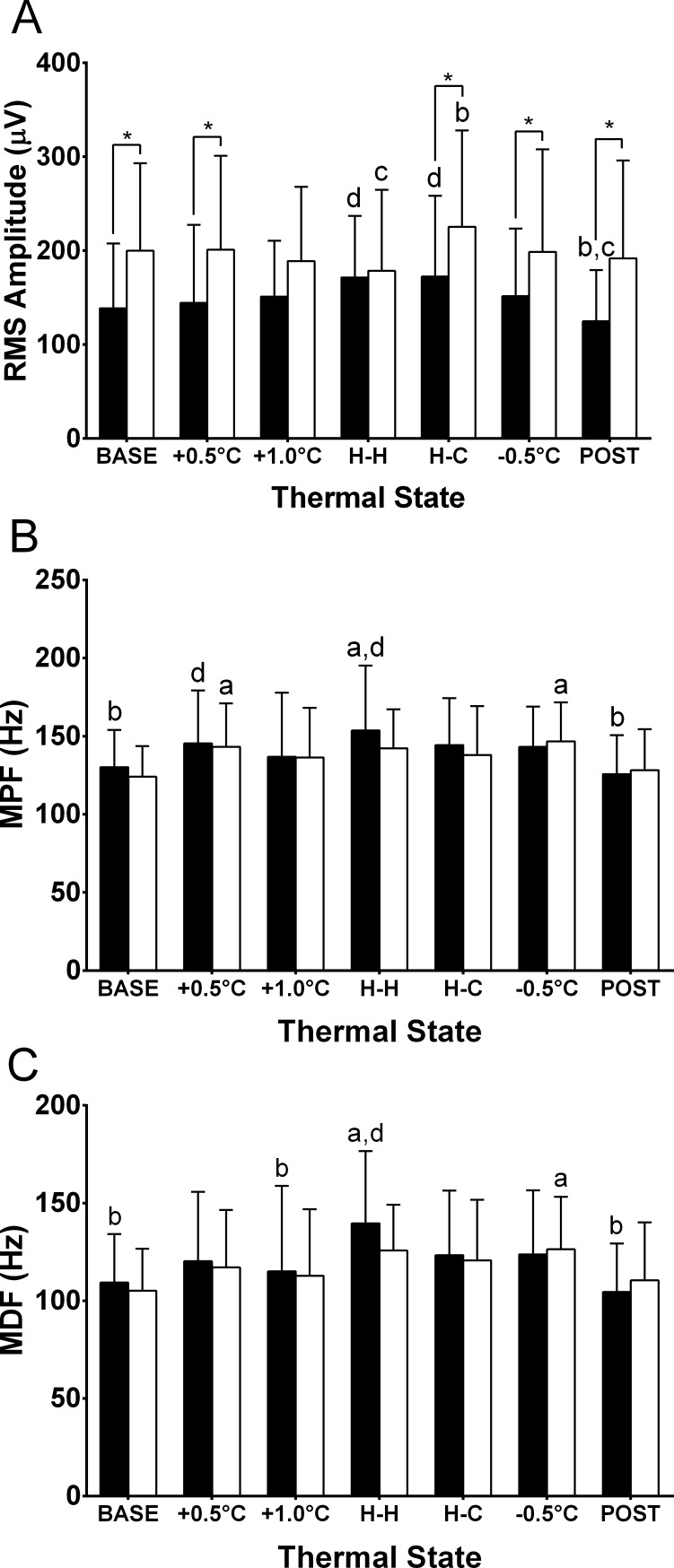
**Electromyographic responses of root-mean-square amplitude (RMS; A), mean power frequency (MPF; B) and median power frequency (MDF; C) to passive heating and cooling for the 3-second isometric force (closed bars) and position (open bars) task in 20 participants.** Temperature states: initial T_re_ and ${\bar{T}}_{\mathrm{sk}}$ (BASE); hot T_re_, hot ${\bar{T}}_{\mathrm{sk}}$ (H-H); hot T_re_, cool ${\bar{T}}_{\mathrm{sk}}$ (H-C); and end of the protocol where T_re_ returned to normal and ${\bar{T}}_{\mathrm{sk}}$ was cool (POST). *Force task significantly different from position task (*p* < 0.05). ^a^Significantly different from baseline (BASE). ^b^Significantly different from hot core-hot skin (H-H). ^c^Significantly different from hot core-cool skin(H-C). ^d^Significantly different from end of protocol (POST).

RMS amplitude, mean power frequency (MPF), and median frequency (MDF) for the 3-s force and position task at each 0.5°C T_re_ interval are presented in Fig 4. Trend analysis of the RMS amplitude (Fig 4A) revealed a significant quadratic (*p* < 0.001, *n* = 20) and cubic (*p* = 0.025, *n* = 20) trend for the isometric force task, whereas no significant trend was identified for the position task. A significant T_re_ x contraction interaction was found for RMS amplitude (*p* > 0.010, *n* = 20). Trend analysis of MPF (Fig 4B) and MDF (Fig 4C) for both the isometric force and position tasks revealed a quadratic trend (all *p* ≤ 0.043, *n* = 20) with no other significant trend identified. No T_re_ x contraction interaction effects were observed for MPF or MDF (*p* ≥ 0.356, *n* = 20). For the isometric force task, both MPF and MDF were higher at H-H compared to BASE (both *p* ≤ 0.002, *n* = 20).

### Sustained 1-min force and position task

**Fig 5:**
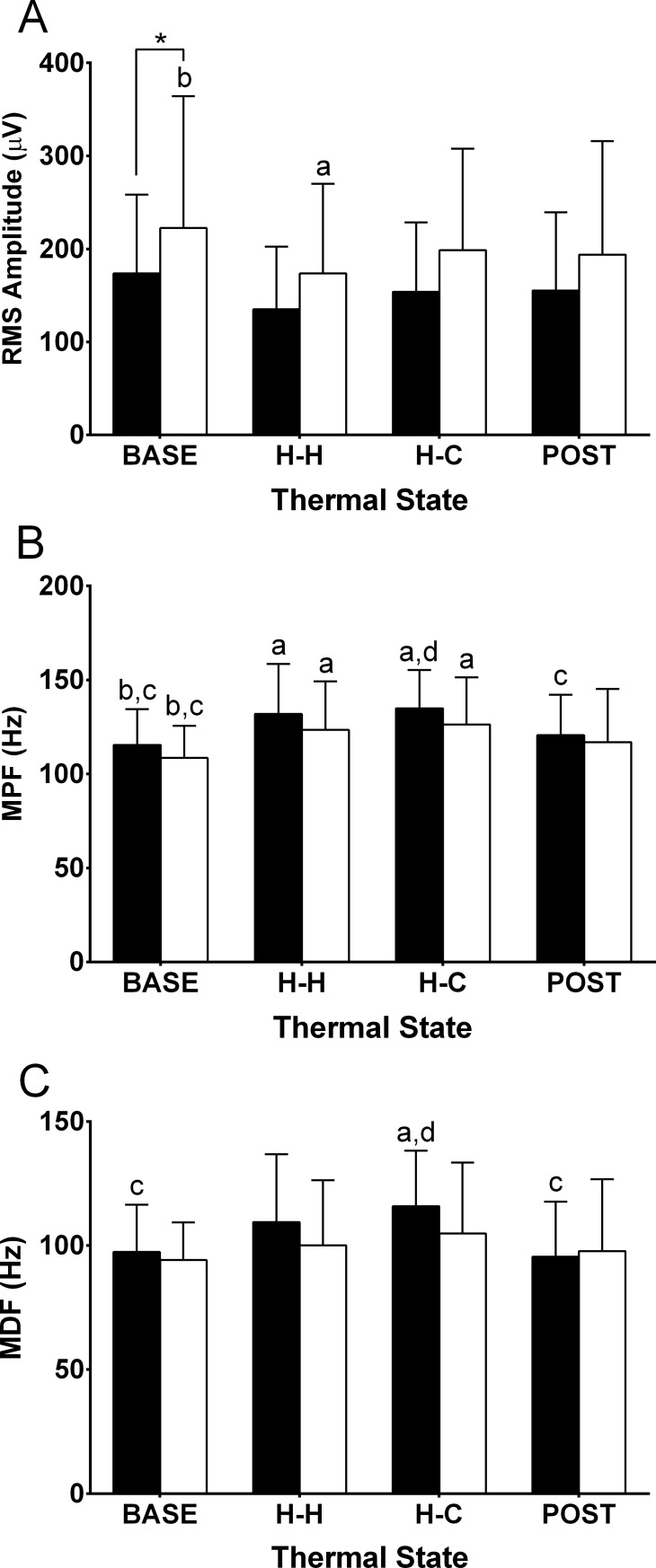
**Electromyographic responses of root-mean-square amplitude (RMS; A), mean power frequency (MPF; B) and median power frequency (MDF; C) to passive heating and cooling for the 1-minute isometric force (black bars) and position (open bars) task in 18 participants.** Temperature states: initial T_re_ and ${\bar{T}}_{\mathrm{sk}}$ (BASE); hot T_re_, hot ${\bar{T}}_{\mathrm{sk}}$ (H-H); hot T_re_, cool ${\bar{T}}_{\mathrm{sk}}$ (H-C); and end of the protocol where T_re_ returned to normal and ${\bar{T}}_{\mathrm{sk}}$ was cool (POST). ^a^Significantly different from baseline (BASE). ^b^Significantly different from hot core-hot skin (H-H). ^c^Significantly different from hot core-cool skin(H-C). ^d^Significantly different from end of protocol (POST).

RMS amplitude, MPF, and MDF activity for the 1-min sustained tasks are presented in Fig 5. Trend analysis for RMS amplitude (Fig 5A) revealed no significant trends for the isometric force task, whereas a significant cubic trend was identified for the position task (*p* = 0.009, *n* = 18). RMS amplitude of the position task was significantly higher at BASE compared to the isometric force task (*p* = 0.038, *n* = 18), but was not significant for all other time points. RMS amplitude for the position task was lower at H-H compared to BASE (*p* = 0.038, *n* = 18). Both contraction types demonstrated a significant quadratic trend (*p* ≤ 0.004, *n* = 18) for MPF (Fig 5B); whereas only the isometric force task demonstrated a significant quadratic trend (*p* < 0.001, *n* = 18) for MDF (Fig 5C). For the 1-min task, MPF was significantly higher with hyperthermia (H-H, H-C) for both the force and position tasks (*p* ≤ 0.003, *n* = 18) compared to BASE. MDF was higher at H-C compared to BASE and POST (both *p* ≤ 0.022, *n* = 18) for the isometric force task.

## Discussion

This study compared the relative contributions of core and skin thermal afferents on sEMG responses to isometric force and position contractions during passive hyperthermia. A passive heating and cooling protocol permitted the investigation of sEMG activity from normothermia (~37.1°C) to a hyperthermia of ≥1.5°C at ~0.5°C intervals, and then again at 0.5°C intervals during passive cooling back to 37.8°C; thus, comparisons could be made at similar core temperatures with both warm and cool skin temperatures. Similar maximal torque production and sensorimotor responses before and following the passive heating and cooling protocol suggest that any changes to sEMG responses would not be a result of central or peripheral fatigue accumulating over the protocol, but more so directly influenced by the thermal manipulations of T_re_ and ${\bar{T}}_{\mathrm{sk}}$. This is further supported by the observed increase in MPF with elevated core temperatures during the sustained 1-min contractions. During fatigue, it has been shown that MPF decreases [25,35,36], yet this study revealed the opposite results. Also, similar V-wave peak-to-peak amplitudes during the preliminary analysis on a subset of participants revealed that the central outflow to the muscle was similar between contraction types, supporting the hypothesis that any central changes between contractions types were a result of thermal manipulations. A novel aspect of this study was the use of a submaximal isometric position task as a more representative model of dynamic muscular contractions compared to maximal isometric force tasks. The primary finding was that the sEMG RMS amplitude responded differently to progressive hyperthermia between isometric force and position tasks. Secondly, it appears that T_re_ and ${\bar{T}}_{\mathrm{sk}}$ thermal afferents were the main contributors to changes in the isometric force task and position task RMS amplitude, respectively. These data support central nervous system activation being largely driven by elevations in core temperature during isometric contractions [4–7], whereas decreases in skin temperature drive decreases in torque output during isokinetic contractions [22].

### Isometric force task

RMS amplitude was used as an indirect measure of motor unit recruitment, firing rate, and conduction velocity [25]. In this study, trend analysis of the 3 s task revealed a dominant quadratic pattern of increasing amplitude with passive hyperthermia, supporting previous work where changes in voluntary activation using interpolated twitches decreased in a quadratic pattern with increases in core temperature during a 3–5 s MVC [6]. Todd et al. [7] reported that higher muscle temperature was associated with a ~20% increase in peak relaxation rate and consequently, higher motor unit firing rates would be required to achieve fusion force. Thus, the progressive increase in RMS amplitude with increasing T_re_ during our 3-s submaximal isometric force task may reflect temperature-induced changes in muscle properties eliciting a compensatory increase in the recruitment and firing rates of motor units in order to maintain the target level of force [25,35,37].

MPF and MDF for the isometric force task was elevated with hyperthermia. This suggests a shift towards higher frequencies driven by a combination of both high core and high skin temperatures, possibly from an increase in muscle and nerve conduction velocity [20,24]. This increase could be attributed to sodium and potassium channels opening and closing at faster rates, causing a decreased amplitude and duration of motor unit action potentials [21]. However, the combination of increases in power spectrum parameters along with RMS amplitude may also reflect an increase in relative force output at higher core temperatures [38]. In this study, workload was held constant throughout at 60% of MVC at baseline. If MVC output decreases at high T_re_, the relative workload would have progressively increased during each stage of the heating phase. This would cause an increase in the recruitment of larger diameter motor units as force level is increased, resulting in increases in both motor unit amplitude and firing frequency [26,38].

### Position task

Interestingly, during brief 3-s contractions, RMS amplitude did not change during progressive passive hyperthermia, yet decreases in ${\bar{T}}_{\mathrm{sk}}$ upon the initiation of skin cooling–despite elevated core temperature–elicited increases in RMS amplitude. This concurs with Cheung and Sleivert [22], who showed decreases in isokinetic force output when the skin was rapidly cooled while core temperature remained elevated, and is fundamentally distinct from the isometric force task. The increase in amplitude was also supported by Winkel and Jorgensen [28], who reported a two-fold increase in sEMG amplitude during dynamic contractions with skin cooling compared to warm skin when core temperature remained at a thermoneutral baseline. The differences in the pattern of the RMS amplitudes during isometric force and position tasks in response to core and skin temperature changes highlight the distinction between thermal afferents that drive different modalities of muscular contraction.

MPF and MDF did not significantly change throughout the heating protocol for the 3-s position task. The only observable difference was an increase in MPF and MDF partway through the cooling phase (-0.5°C) compared to baseline, suggesting that the contribution of frequencies to the sEMG signal were relatively constant until this point, whereupon there was a transient shift to higher frequencies. A potential explanation for the trend in spectral parameters observed for the position task is conduction velocity. Although not measured in this study, Rutkove et al. [20] reported that with cooler skin temperatures, conduction velocity is slightly decreased and ion-gated channels remains open for longer periods of time. This allows the increase in ion flux through the channel, producing a larger depolarization and action potential. This change in conduction velocity with cooling of the skin may explain the transient shift towards higher frequencies.

### Comparing force and position tasks responses to heating

Differences in the sEMG signal between contraction modalities may lie in central control of different types of contractions, and their inherent motor unit characteristics [39]. Ivanova et al. [40] sought to examine motor unit characteristics between isometric and dynamic movements with the same torque-time characteristics. This provided a critical methodological control to isolate central control mechanisms between the two contractions. The authors demonstrated that the motor unit recruitment threshold was lower in dynamic movements than in isometric contractions. Therefore, a potential mechanism underlying the stable sEMG responses of a position task to progressive hyperthermia—reflected by RMS amplitude, MPF, and MDF–is that the lower motor unit recruitment threshold requires less additional motor unit firing to compensate for changes in muscle properties. While core temperature had no impact on neural drive during a position task, the rapid decrease in skin temperature during cooling transiently increased the neural drive required to maintain the submaximal load. This suggests a significant contribution of skin temperature on motor unit recruitment and firing rate required for a position task.

The intramuscular pressure generated during an isometric contraction prevents blood flow to allow for metabolites, interfere with the contractile mechanism, to be cleared [27]. In contrast, blood flow during a dynamic contraction has been shown to be maintained by enhancing the venous return by the contracting muscle and removing metabolic by-products [41]. It has been suggested that blood flow occlusion with isometric contractions could result in motor unit rotation, where the sEMG signal shifts towards higher frequencies as other motor units become impaired by ischemia [42]. This assumption could explain the progressive increase in spectral parameters during the force task and the lack of change during the position task.

### Methodological considerations

Two significant methodological controls in this study enabled the comparison of neuromuscular function between tasks. There were no changes in maximal torque or M_max_ amplitude before and following the protocol, demonstrating that changes to the sEMG signal resulted from thermal manipulations of T_re_ and ${\bar{T}}_{\mathrm{sk}}$ rather than a result of central or peripheral fatigue accumulating over the protocol. This project was done in a single session always in the order of baseline, progressive hyperthermia, and returning to baseline T_re_, thus we cannot exclude the possibility of this order influencing results. However, this progressive heating and cooling protocol has successfully been employed previous by our lab [4,6,17,18,22]. These studies have all demonstrated a significant impairment of maximal voluntary torque with similar levels of passive hyperthermia as in the present study. To avoid over-stressing participants and overall neuromuscular fatigue, we did not perform maximal testing at peak hyperthermia; thus, it remains possible that we did not cause significant hyperthermic impairment. Secondly, matching the load between the isometric force and position task allowed the comparison of central control mechanisms of each contraction independent of any changes to the difference in force/length curves between contraction types [39].

One reason for selecting the FCR as the tested muscle was because of its thin and superficial nature, such that there can be a reasonable assumption of a correlation between skin and muscle temperature. However, as we did not directly measure muscle temperature, and also did not directly quantify muscle or nerve conduction velocity, the potential effects of thermally-induced velocity changes on our frequency data remain speculative.

A young population (22–29 years old) was tested due to changes in motor unit characteristics with aging, including: lower motor unit discharge rate, decreases in nerve conduction velocity, and a decrease in the number of motor units present [43]. Therefore, results from this study may not be generalizable to an older population. It could be assumed that the increase in neural drive to compensate for changes in muscle properties during heat stress may be dampened in older individuals due to these changes in motor unit properties.

Peripheral vasodilation and electrode location during passive heat stress increases the distance between the muscle and the sEMG electrode [29], and could potentially act as additional tissue filtering, dampening the signal to lower frequencies recorded at the surface of the skin. Spectral analysis during hyperthermia may need to be interpreted with caution as the frequency spectrum may be underestimated with additional tissue filtering. However, the differences between contraction type as core remained elevated with both hot and cool skin is a strong indication that changes to the sEMG amplitude and spectral parameters were a result of the experimental manipulations.

## Conclusions

This study demonstrated that core temperature was the primary thermal afferent influencing sEMG amplitude during a brief isometric force task, independent of changes to skin temperature. In contrast, sEMG amplitude during a position task were not affected by core temperature increases, but rapidly lowering skin temperature increased sEMG amplitude. These findings suggest that the neural drive to the muscle during hyperthermia is dependent on both the task being performed and the relative role of core and skin thermal afferents. Importantly, this suggests that, for future studies investigating the role of hyperthermia on neuromuscular function during whole-body dynamic exercise, it is critical to move beyond reliance on maximal isometric force contractions models and develop and utilize neuromuscular testing protocols that more closely reflect the unique nature of dynamic contraction.

## Supporting information

S1 File Supplementary individual thermal and neuromuscular data. (XLSX)
